# Supplementary material for: Ultraconserved Elements and Machine Learning Classifiers Enable Robust Phylogenetics and Taxonomy in Model and Non‐Model Nematodes
Source: Mol Ecol Resour. 2025 Oct 8;25(8):e70046. doi: 10.1111/1755-0998.70046 (PMC12550484; doi:10.1111/1755-0998.70046)
Supplement: Supplementary file 1 — Data S1: Supporting Information. [file MEN-25-e70046-s001.pdf]

A Supplementary Information

Annex

A.1 Bait set design

**Sample Name**	**Accession Number**
LJ2406	SRX18142758
LJ2414	SRX18142759
LJ2400	SRX18142756
P. superbus	SRX2562390
PS1579	SRX2562526
PS1159	SRX2562411
ES5	SRX2562528

Table 3: Re-sequencing data accession numbers of the different strains tested for the bait set design of the Panagrolaimidae family

**Genera**	**GenBank Accession**
Panagrellus	GCA_341325.1
Halicephalobus	GCA_9193035.1
Halicephalobus	GCA_9761265.1
Panagrolaimus	GCA_28622995.1
Acrobeloides	GCA_34698545.1
Acrobeloides	GCA_34699885.1
Acrobeloides	GCA_34700925.1
Panagrolaimus	GCA_963922195.1
Turbatrix	GCA_963969345.1
Panagrolaimus	GCA_964035985.1
Propanagrolaimus	GCA_964059925.1
Neocephalobus	GCA_964187885.1
Acrobeloides	GCA_964212105.1
Propanagrolaimus	GCA_964245515.1
Panagrolaimus	GCA_964249645.1

Table 4: Genera and corresponding GenBank accession numbers for phylogenetic reconstruction of Panagrolaimidae family.

Species name or strain code	Isolation Origin
Acrobeloides cf. *guoghiensis* ARO.22.05	Atacama Desert, Chile
Panagrolaimus sp. ALT.22.04	Atacama Desert, Chile
Panagrolaimus sp. ALT.22.08	Atacama Desert, Chile
Panagrolaimus sp. JU2834	Kelaat Sraghna, Maroc
Panagrolaimus sp. PS1579	California, United States
Panagrolaimus superbus	Surtsey Island, Iceland
Panagrolaimus sp. JU1358	Kerala, India
Panagrolaimus sp. JU1366	Tamil Nadu, India
Panagrolaimus sp. JU1371	Pondicherry, India
Panagrolaimus sp. JU1387	La Reunión, France
Panagrolaimus sp. JU1645	Santo Antao Island, Cape Verde
Panagrolaimus sp. JU1646	Santiago Island, Cape Verde
Panagrolaimus sp. JU2071	Europa Island, France
Panagrolaimus sp. JU2212	Hüschul uul, Mongolia
Panagrolaimus sp. JU2852	Cordoba, Argentina
Panagrolaimus sp. JU2885	Lofoten Islands, Norway
Acrobeloides tricornis PAP.22.17	Atacama Desert, Chile
Panagrolaimus sp. PAP.22.29	Atacama Desert, Chile
Panagrolaimus sp. PAP.22.38	Atacama Desert, Chile
Panagrolaimus sp. PAP.22.39	Atacama Desert, Chile

Table 5: Species and strain names and isolation area of cultures used for generating target capture data in this study.

Strain or species	Concentration (ng/µl)	260/280	260/230
Acrobeloides tricornis PAP.22.17	15	1.87	0.76
Acrobeloides cf. *guoghiensis* ARO.22.05	9	1.72	0.54
Panagrolaimus sp. JU2852	33.1	1.98	1.04
Panagrolaimus sp. JU2885	51.3	1.81	1.21
Panagrolaimus sp. PAP.22.29	39.8	1.86	0.08
Panagrolaimus sp. PAP.22.38	11.3	1.83	0.07
Panagrolaimus sp. PAP.22.39	32.8	1.93	1.35
Panagrolaimus sp. JU1645	35.5	1.92	0.18
Panagrolaimus sp. JU1366	12.6	1.95	0.09
Panagrolaimus sp. JU1646	30.4	1.71	0.38
Panagrolaimus sp. JU1371	33.3	1.93	0.64
Panagrolaimus sp. JU2071	57.6	1.92	1.37
Panagrolaimus sp. JU1358	28.7	1.75	0.41
Panagrolaimus sp. JU1387	41.9	1.93	1.03
Panagrolaimus superbus	78.1	1.95	1.06
Panagrolaimus sp. ES5	66.3	1.95	1.24
Panagrolaimus sp. JU2212	39.3	1.97	0.72
Panagrolaimus sp. JU2834	52.9	1.76	0.78
Panagrolaimus sp. PS1579	69.6	1.91	0.88
Panagrolaimus sp. ALT.22.08	11	1.65	0.15
Panagrolaimus sp. ALT.22.04	66.7	1.91	2.53

Table 6: Species and strains DNA concentration with corresponding 260/280 and 260/230 ratios measured using Nanodrop.

A.2 Morphological description of *Neocephalobus halophilus* BSS8 strain, previously referred to as *Panagrolaimus detritophagus* BSS8

Adult: Body nearly straight when killed by heat, posterior end more curved in males, but not J-shaped. Cuticle finely annulated. Lateral alae with three incisures extending to phasmid. Lip region continuous with body contour; lips partially merged in three pairs, one dorsal and two ventrolateral, although six individual tips are discernible at highest magnification. Labial and cephalic sensilla and amphids are indistinct. Stoma anisomorphic, with distinct metastegostomatal dorsal tooth. Pharynx distinctly subdivided into corpus, isthmus and basal bulb: pharyngeal corpus broad cylindrical; isthmus much narrower, demarcated by a break in muscular tissue; basal bulb oval, with distinct grinder. Nerve ring and deirid at level of isthmus. Excretory pore location varies between base of corpus to anterior part of basal bulb level (supplementary figures 7 and 8).

Female: Reproductive system monodelphic, prodelphic, located on the right side of the intestine (dextral); ovary straight. Oviduct very short, about one- half of the corresponding body diameter. Spermatheca axial, well developed. Postvulval uterine sac equal to the corresponding body diameter. Vagina straight, vulval lips protruding. Rectum short. Tail straight, elongate-conoid. Phasmids located at anterior third of tail length (supplementary figures 6 and 9).

Male: Reproductive system monorchic, with testis reflexed ventrad. Spicules slender, paired and symmetrical, weakly curved ventrad, with oval manubrium and gradually narrowing shaft. Gubernaculum plate-like. Genital papillae distributed as follows: one pair of subventral precloacal papilliform sensilla located anterior to spicules, single unpaired and much larger precloacal papilliform sensillum located at level of spicule manubrium, one pair of subventral precloacal papilliform sensilla located just anterior to clocal opening, one pair of subventral postcloacal papilliform sensilla located between cloacal opening and phasmid, one pair of subventral postcloacal papilliform sensilla and one pair of subdorsal postcloacal papilliform sensilla located near phasmid, at about the middle of tail length. Tail straight, elongate-conoid (supplementary figure 10).

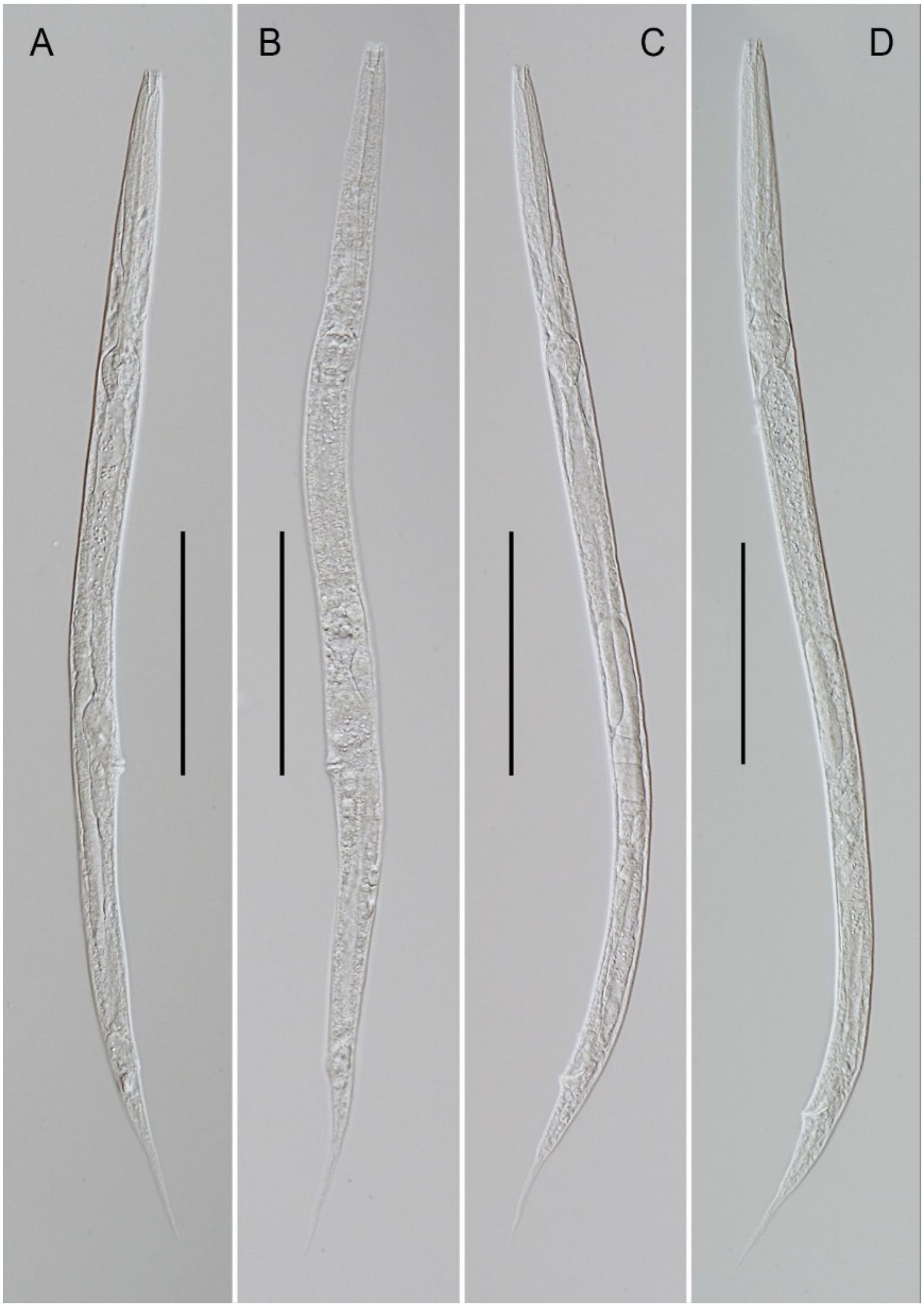

Figure 6: *Neocephalobus halophilus* Paetzold, 1958 (strain BSS8). Entire female (A-B) and male (C-D). Scale bars: A-D = 100 μm.

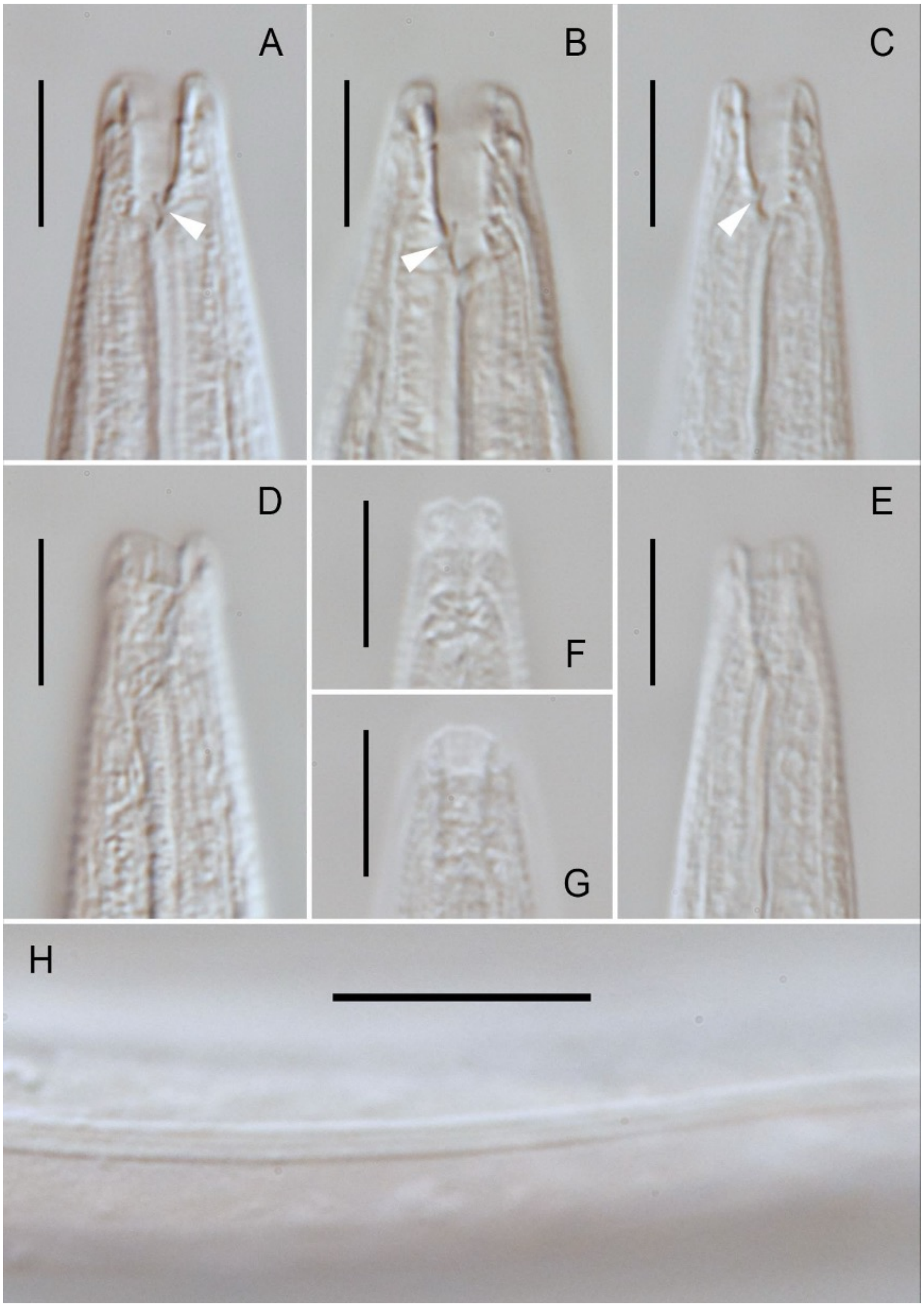

Figure 7: *Neocephalobus halophilus* Paetzold, 1958 (strain BSS8). A-C: Anterior body end, median section (dorsal to the right in A and to the left in B-C, arrow points to the dorsal tooth in A-C); D-E: Lateral view of the labial region (dorsal to the right in D and to the left in E); F: Ventral view of the labial region; G: Dorsal view of the labial region; H: Lateral alae. Scale bars: A-G = 10 μm, H = 20 μm.

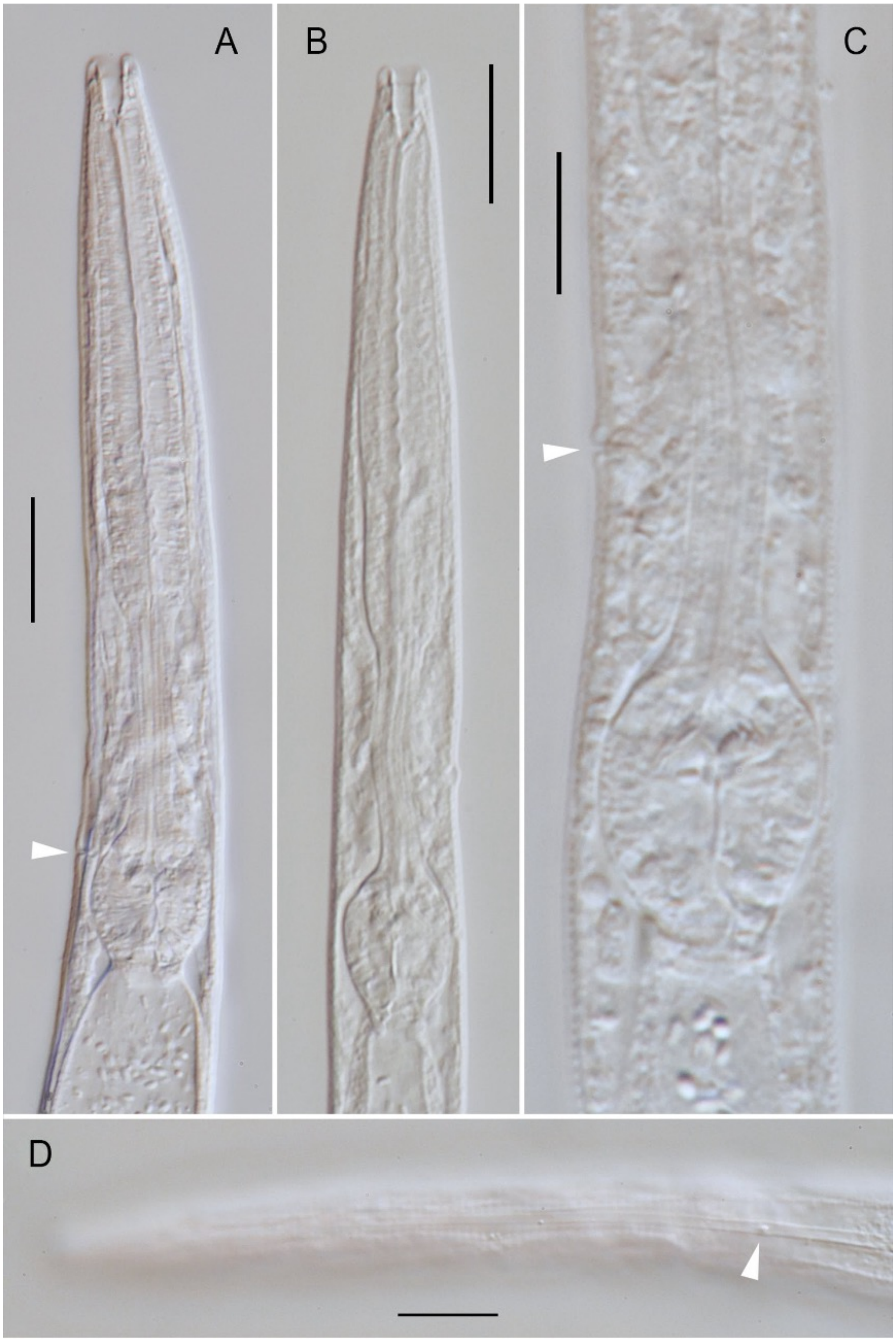

Figure 8: *Neocephalobus halophilus* Paetzold, 1958 (strain BSS8). A-B: Pharyngeal region, median section, showing excretory pore in A (arrow). C: Isthmus and basal bulb, showing excretory pore (arrow). D: Surface view of the pharyngeal region, showing deirid (arrow). Scale bars: A-D = 20 μm

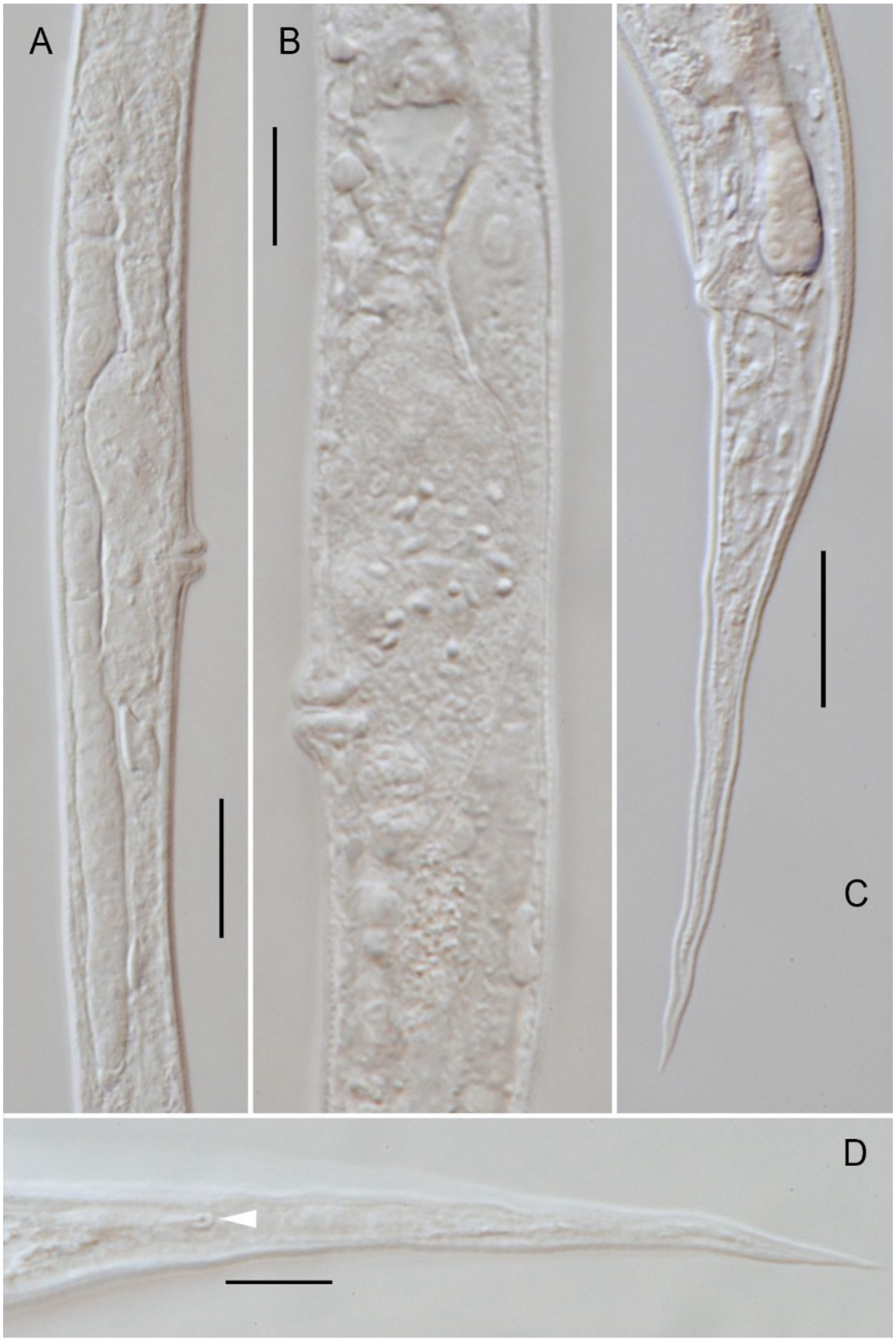

Figure 9: *Neocephalobus halophilus* Paetzold, 1958 (strain BSS8): A. Entire female reproductive system. B: Part of the female reproductive system showing uterus, vulva and post-vulval uterine sac. C: Female tail. D: Phasmid (arrow). Scale bars: A-D = 20 μm.

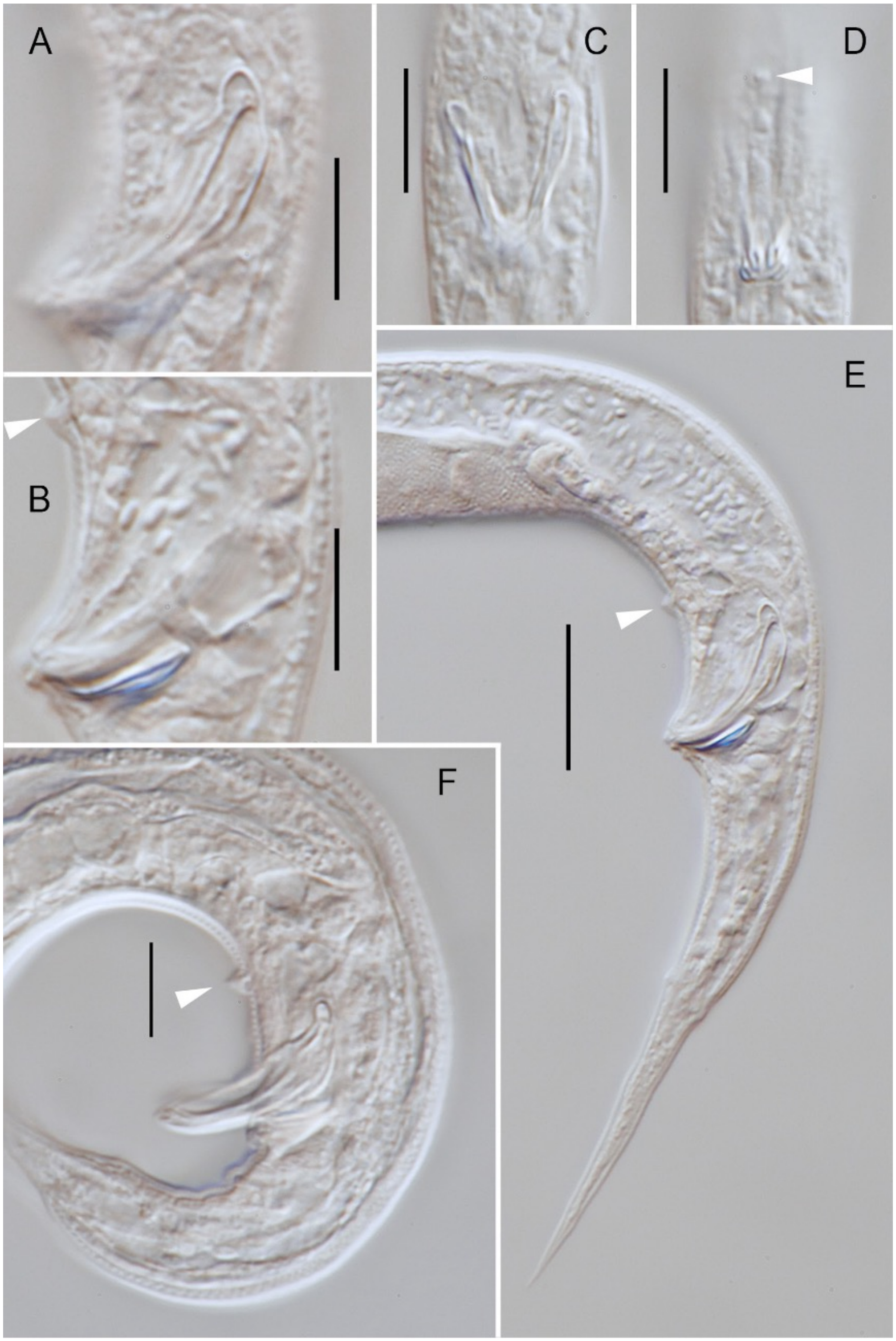

Figure 10: *Neocephalobus halophilus* Paetzold, 1958 (strain BSS8). Lateral (A-B) and ventral (C-D) views of cloacal region. E-F: Lateral view of caudal region. Arrow points to midventral precloacal papilliform sensillum in B and D-F. Scale bars: A-D = 10μm, E-F = 20 μm.

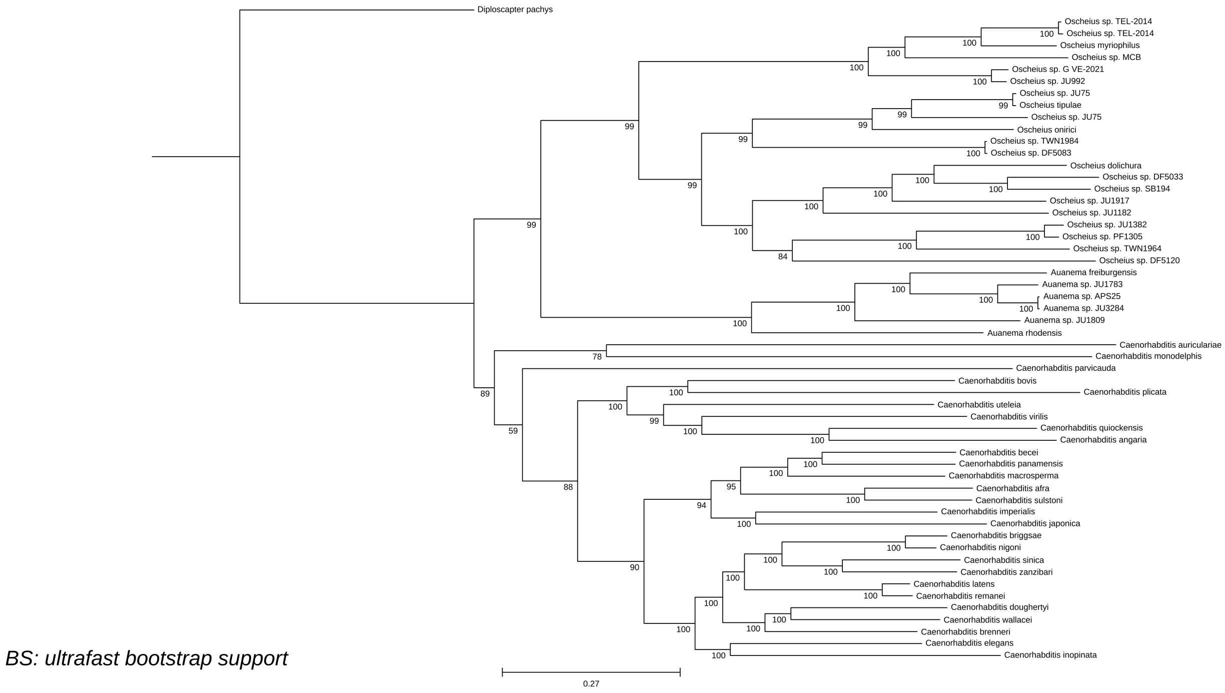

Figure 11: Phylogenetic reconstruction of the Rhabditidae family based on UCEs. *Diploscapter coronatus* is excluded due to very long branch (possibly LBA) and incorrect placing given the low amount of UCEs harvested in the genome. This reconstrucction is based in a 65% occupancy matrix based on 215 alignments.

**Species**	Neocephalobus halophilus BSS8	Neocephalobus halophilus BSS8	Neocephalobus halophilus	Neocephalobus aberrans	Panagrolaimus orthomici	Korentchenko, 1992	Galleria mellonella	Bark beetles
**Reference**	Recent	Boström, 1988 (pop. II)	Paetzold, 1958	Steiner, 1929	Bhat et al., 2025	–	–	–
Habitat	Culture	Gull nest	Culture	Soil	Culture	Feces	Galleria mellonella	Bark beetles
Number of individuals	9	5	20	10	20	?	15	30
Body length	657±47 (609–744)	731±14 (705–765)	759±15 (635–860)	477–605	775–1070	?	444±15 (430–560)	530±12 (436–640)
a	21.6±1.7 (18.2–24.3)	25±1 (23–27)	20±0.4 (16–23)	19.8–23.8	15.9–21.5	?	19.8±1.9 (17.7–21.5)	16.5±0.7 (12.1–23.6)
b	6.1±0.3 (5.7–6.8)	5.4±0.1 (5.3–5.7)	6.2±0.1 (5.3–6.8)	4.1–4.8	5.0–6.9	?	3.9±0.1 (3.8–4.0)	5.4±0.1 (4.6–6.4)
c	7.6±0.7 (6.3–8.4)	8.5±0.3 (8–9)	10±0.1 (9–11)	6.2–7.7	7.8–10	?	9.5±0.5 (9.0–10.2)	8.3±0.1 (7.5–9.5)
c	5.0±0.6 (4.5–6.4)	4.8±0.1 (4.6–5.2)	4.5±0.1 (3.1–5.3)	?	?	?	3.4±0.1 (3.3–3.5)	?
V (%)	55±2 (52–57)	60±0.4 (58–60)	58±0.2 (56–60)	54–59	55.2–58.7	?	6.3±1.1 (5–8)	57.3±0.2 (55.2–59)
Lip region width	4.4±0.4 (4–5.5)	?	?	?	?	?	10.6±1.2 (10–12)	?
Stoma length	8.9±0.7 (8.0–10.5)	7–10	7–10	?	?	?	?	7.7±0.2 (6–9)
Corpus length	75±7 (67–86)	?	?	?	?	?	27±2.0 (25–29)	?
Isthmus length	32±3 (25–36)	?	?	?	?	?	444±15 (430–560)	?
Basal bulb	22±1 (21–25)	21–22	19–24	?	?	?	15.2±2.6 (13–18)	?
Pharyngeal region length	139±8 (132–155)	135±2 (132–142)	123±1 (114–135)	110–128	?	?	113.3±7.6 (108–122)	99±1 (93–110)
Excretory pore from ant. end	97±5 (90–106)	?	?	?	?	?	93.3±4.2 (90–98)	?
Body width at mid-body	31±2 (28–34)	30±1 (26–33)	39±2 (28–50)	23–26	?	?	22.7±3.1 (20–26)	33±2 (19–49)
Body width at anus	18±1 (16–19)	?	?	?	?	?	13.6±1.2 (13–15)	?
Tail length	87±8 (77–105)	83±3 (76–91)	79±1 (69–89)	65–91	?	?	46.7±4.0 (43–51)	64±2 (50–81)
Post-vulval uterine sac	?	25–42	30–50	?	?	?	11.3±2.3 (10–14)	?
Post-vulval uterine sac / BW	?	0.9–1.3	0.7–1.2	?	?	?	0.4–0.6	?

Table 7: Supplementary Table 7: Morphometrics of *Neocephalobus halophilus* Paetzold, 1958 (strain BSS8), and closely related species (females). Values are means ± standard deviation followed by ranges in parentheses. "?" denotes unavailable data.

**Species**	Neocephalobus halophilus BSS8	Neocephalobus halophilus BSS8	Neocephalobus halophilus	Neocephalobus aberrans	Panagrolaimus orthomici
**Reference**	Recent	Boström, 1988 (pop. II)	Paetzold, 1958	Steiner, 1929	Bhat et al., 2025
Habitat	Culture	Gull nest	Culture	Soil	Culture
Number of individuals	9	6	20	10	20
Body length	604±37 (539–656)	655±19 (600–715)	716±8 (660–795)	435–538	686–880
a	24.5±1.8 (22.6–27.9)	23±1 (20–25)	23±0.4 (19–26)	21.8–29.9	15.8–24.7
b	6.0±0.4 (5.5–6.6)	5.4±0.1 (5.2–5.7)	6.1±0.1 (5.4–6.5)	3.9–4.4	4.8–6.4
c	9.2±0.5 (8.3–9.7)	9±0.3 (8–10)	11±0.2 (9–12)	6.9–7.8	9.4–11.4
c	3.7±0.1 (3.6–3.9)	3.9±0.1 (3.5–4.0)	3.7±0.1 (3.2–4.4)	?	?
Lip region width	3.8±0.3 (3.5–4.0)	?	?	?	?
Stoma length	8.8±0.5 (8.0–9.5)	6–9	6–9	?	?
Corpus length	67±4 (61–71)	?	?	?	?
Isthmus length	34±2 (32–37)	?	?	?	?
Basal bulb	21±0.5 (20–21)	19–21	17–21	?	?
Pharyngeal region length	130±4 (123–136)	121±2 (116–129)	118±1 (109–129)	100–125	?
Excretory pore from ant. end	88±6 (79–96)	?	?	?	?
Body width at mid-body	25±2 (20–26)	28±1 (26–33)	32±0.6 (28–36)	16–21	?
Body width at cloaca	18±1 (16–18)	?	?	?	?
Tail length	66±2 (63–69)	64±1 (59–66)	68±1 (61–73)	60–72	?
Spicule length	17±1 (16–19)	23±0.3 (22–24)	24±0.4 (22–27)	16.5–18	19–22.5
Gubernaculum length	9±1 (7–10)	10±0.2 (10–11)	10.5±0.2 (9–12)	?	?
Male papilla	sv.mv/sv/sv.sv.ph.sd	sv.mv/sv/sl.sv.sv.ph.sd	sv.mv/sv/sv.sv.ph.sd	sv.mv.sv/sd.sv.sv.ph.sd	sv.mv.sv/sl.sv.ph.sd.sd.sv

Table 8: Supplementary Table 8: Morphometrics of *Neocephalobus halophilus* Paetzold, 1958 (strain BSS8), and closely related species (males). Values are means ± standard deviation followed by ranges in parentheses. "?" denotes unavailable data.

Genera	GenBank Accession
Oscheius	GCA_932521025.1
Oscheius	GCA_932521405.1
Oscheius	GCA_932521415.1
Oscheius	GCA_932521035.1
Oscheius	GCA_964036215.1
Oscheius	GCA_964057215.1
Oscheius	GCA_037178975.1
Oscheius	GCA_037178855.1
Oscheius	GCA_022343465.1
Oscheius	GCA_000934875.1
Oscheius	GCA_001513535.1
Oscheius	GCA_013425905.1
Oscheius	GCA_964264085.1
Oscheius	GCA_964264175.1
Oscheius	GCA_964264275.1
Oscheius	GCA_964264245.1
Oscheius	GCA_964261225.1
Oscheius	GCA_964264255.1
Oscheius	GCA_964261325.1
Oscheius	GCA_964261265.1
Oscheius	GCA_964264185.1
Oscheius	GCA_037179065.1
Oscheius	GCA_037178915.1
Oscheius	GCA_037179185.1
Oscheius	GCA_037178995.1
Oscheius	GCA_037179095.1
Oscheius	GCA_037178805.1
Oscheius	GCA_037178875.1
Oscheius	GCA_037179085.1
Oscheius	GCA_037179045.1
Oscheius	GCA_037179165.1
Oscheius	GCA_037178765.1
Oscheius	GCA_037178905.1
Oscheius	GCA_037178795.1
Oscheius	GCA_037178965.1
Oscheius	GCA_037178985.1
Oscheius	GCA_037178755.1
Oscheius	GCA_037179145.1
Oscheius	GCA_037178945.1
Oscheius	GCA_037179075.1
Oscheius	GCA_900184235.1
Oscheius	GCA_022343475.1
Oscheius	GCA_022343505.1
Oscheius	GCA_964036205.1
Oscheius	GCA_964264195.1
Oscheius	GCA_964263735.1
Oscheius	GCA_964263365.1
Oscheius	GCA_964057235.1
Oscheius	GCA_964263805.1
Oscheius	GCA_001630785.1
Oscheius	GCA_964263935.1
Oscheius	GCA_964264165.1

Table 9: *Oscheius* GenBank accession numbers — Rhabditidae family.

**Genera**	**GenBank Accession**
Diploscapter	GCA_964036155.1
Diploscapter	GCA_002287525.1
Diploscapter	GCA_002207785.1
Diploscapter	GCA_964036175.1

Table 10: *Diploscapter* GenBank accession numbers - Rhabditidae family.

Genera	GenBank Accession	Genera	GenBank Accession
Caenorhabditis	GCA_000002985.3	*Caenorhabditis*	GCA_963921115.1
Caenorhabditis	GCF_000002985.6	*Caenorhabditis*	GCA_016989365.1
Caenorhabditis	GCA_003052745.1	*Caenorhabditis*	GCA_016989105.1
Caenorhabditis	GCA_027920645.1	*Caenorhabditis*	GCA_016989455.1
Caenorhabditis	GCA_043792875.1	*Caenorhabditis*	GCA_016989115.1
Caenorhabditis	GCA_963570955.1	*Caenorhabditis*	GCA_016989145.1
Caenorhabditis	GCA_963572205.1	*Caenorhabditis*	GCA_963921025.1
Caenorhabditis	GCA_963966625.1	*Caenorhabditis*	GCA_016989505.1
Caenorhabditis	GCA_963572245.1	*Caenorhabditis*	GCA_016989275.1
Caenorhabditis	GCA_000004555.3	*Caenorhabditis*	GCA_016989385.1
Caenorhabditis	GCF_000004555.2	*Caenorhabditis*	GCA_963921155.1
Caenorhabditis	GCA_010183535.1	*Caenorhabditis*	GCA_963921015.1
Caenorhabditis	GCF_010183535.1	*Caenorhabditis*	GCA_016989095.1
Caenorhabditis	GCA_963932045.1	*Caenorhabditis*	GCA_963921145.1
Caenorhabditis	GCA_964036135.1	*Caenorhabditis*	GCA_963921045.1
Caenorhabditis	GCA_964213925.1	*Caenorhabditis*	GCA_963921005.1
Caenorhabditis	GCA_963572235.1	*Caenorhabditis*	GCA_963921065.1
Caenorhabditis	GCA_963932035.1	*Caenorhabditis*	GCA_963921085.1
Caenorhabditis	GCA_963573275.1	*Caenorhabditis*	GCA_013403715.1
Caenorhabditis	GCA_964197825.1	*Caenorhabditis*	GCA_963920985.1
Caenorhabditis	GCA_963931815.1	*Caenorhabditis*	GCA_016989285.1
Caenorhabditis	GCA_964036255.1	*Caenorhabditis*	GCA_016989125.1
Caenorhabditis	GCA_963572285.1	*Caenorhabditis*	GCA_963920965.1
Caenorhabditis	GCA_963572265.1	*Caenorhabditis*	GCA_963921125.1
Caenorhabditis	GCA_963932285.1	*Caenorhabditis*	GCA_963921095.1
Caenorhabditis	GCA_002259235.3	*Caenorhabditis*	GCA_027422445.1
Caenorhabditis	GCA_964198105.1	*Caenorhabditis*	GCA_027422425.1
Caenorhabditis	GCA_963966605.1	*Caenorhabditis*	GCA_033458355.1
Caenorhabditis	GCA_963978915.1	*Caenorhabditis*	GCA_033458345.1
Caenorhabditis	GCA_900536315.3	*Caenorhabditis*	GCA_030248285.1
Caenorhabditis	GCA_902829315.1	*Caenorhabditis*	GCA_018136805.1
Caenorhabditis	GCA_946814055.1	*Caenorhabditis*	GCA_018136875.1
Caenorhabditis	GCA_904845305.1	*Caenorhabditis*	GCA_018136835.1
Caenorhabditis	GCA_900883565.2	*Caenorhabditis*	GCA_018136795.1
Caenorhabditis	GCA_900536305.3	*Caenorhabditis*	GCA_000939815.1
Caenorhabditis	GCA_900536415.3	*Caenorhabditis*	GCA_037024025.1
Caenorhabditis	GCA_900536285.3	*Caenorhabditis*	GCA_037024065.1
Caenorhabditis	GCA_900536295.3	*Caenorhabditis*	GCA_037024035.1
Caenorhabditis	GCA_900536325.3	*Caenorhabditis*	GCA_018990105.1
Caenorhabditis	GCA_900536235.3	*Caenorhabditis*	GCA_963932085.1
Caenorhabditis	GCA_900536345.3	*Caenorhabditis*	GCA_947459285.1
Caenorhabditis	GCA_028201415.1	*Caenorhabditis*	GCA_963572215.1
Caenorhabditis	GCA_028201515.1	*Caenorhabditis*	GCA_964213915.1
Caenorhabditis	GCA_020450165.1	*Caenorhabditis*	GCA_000143925.2
Caenorhabditis	GCA_004526295.1	*Caenorhabditis*	GCA_000147155.1
Caenorhabditis	GCA_022453885.1	*Caenorhabditis*	GCA_000165025.1
Caenorhabditis	GCA_021491975.1	*Caenorhabditis*	GCA_963932055.1
Caenorhabditis	GCA_029581135.1	*Caenorhabditis*	GCA_963570465.1
Caenorhabditis	GCA_000975215.1	*Caenorhabditis*	GCA_000186765.1
Caenorhabditis	GCA_022984815.1	*Caenorhabditis*	GCA_964036165.1
Caenorhabditis	GCA_039880965.1	*Caenorhabditis*	GCA_963932405.1
Caenorhabditis	GCA_029748435.1	*Caenorhabditis*	GCA_963931775.1
Caenorhabditis	GCA_002259225.3	*Caenorhabditis*	GCA_963572255.1
Caenorhabditis	GCA_001643735.4	*Caenorhabditis*	GCA_964197835.1
Caenorhabditis	GCA_002742825.1	*Caenorhabditis*	GCA_963572295.1
Caenorhabditis	GCA_001643685.2	*Caenorhabditis*	GCA_963572225.1
Caenorhabditis	GCA_016735795.1	*Caenorhabditis*	GCA_963572275.1
Caenorhabditis	GCA_964204685.1	*Caenorhabditis*	GCA_900536275.1
Caenorhabditis	GCA_963921135.1	*Caenorhabditis*	GCA_964204725.1
Caenorhabditis	GCA_963921055.1	*Caenorhabditis*	GCA_963978825.1
Caenorhabditis	GCA_963920975.1	*Caenorhabditis*	GCA_964198135.1
Caenorhabditis	GCA_963920995.1	*Caenorhabditis*	GCA_963572305.1
Caenorhabditis	GCA_016989235.1	*Caenorhabditis*	GCA_963966565.1
Caenorhabditis	GCA_016989295.1	*Caenorhabditis*	GCA_963966575.1
Caenorhabditis	GCA_016989245.1	*Caenorhabditis*	GCA_001483305.2
Caenorhabditis	GCA_963921035.1	*Caenorhabditis*	GCA_900160655.1
Caenorhabditis	GCA_963921075.1	*Caenorhabditis*	GCA_000149515.1
Caenorhabditis	GCA_963921105.1	*Caenorhabditis*	GCF_000149515.1

Table 11: *Caenorhabditis* GenBank accession numbers - Rhabditidae family.

**Genera**	**GenBank Accession**
Auanema	GCA_964057225.1
Auanema	GCA_030370435.1
Auanema	GCA_943334845.2
Auanema	GCA_964264295.1
Auanema	GCA_964263715.1
Auanema	GCA_964263695.1
Auanema	GCA_947366455.1
Auanema	GCA_964057245.1
Auanema	GCA_964263765.1

Table 12: *Auanema* GenBank accession numbers - Rhabditidae family.

A.3 Distribution and characteristics of UCEs in Rhabditidae and Panagrolaimidae

We assessed the distribution of Ultra-Conserved Elements (UCEs) across strains in the families Rhabditidae and Panagrolaimidae by analyzing the total number of UCEs per strain. The results, presented in Figures 13 and 12, reveal considerable variation in UCE counts among strains within each family. Specifically, in Rhabditidae, UCE counts range from 1 to 5700, with a median of 3840, while in Panagrolaimidae, they range from 15 to 1457, with a median of 790.0.

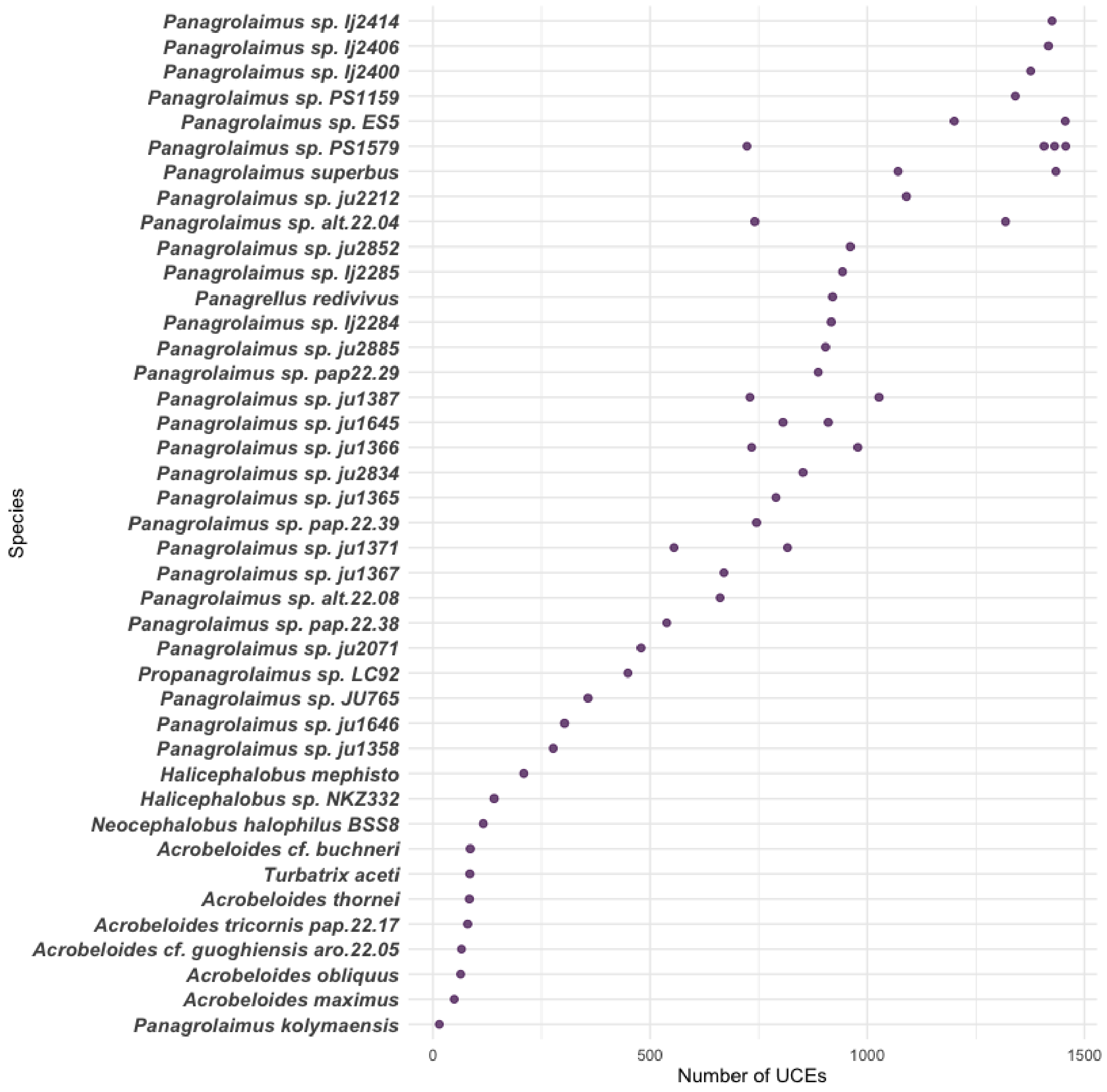

Figure 12: Number of UCEs per species in Panagrolaimidae. Species are sorted from lowest to highest UCE count.

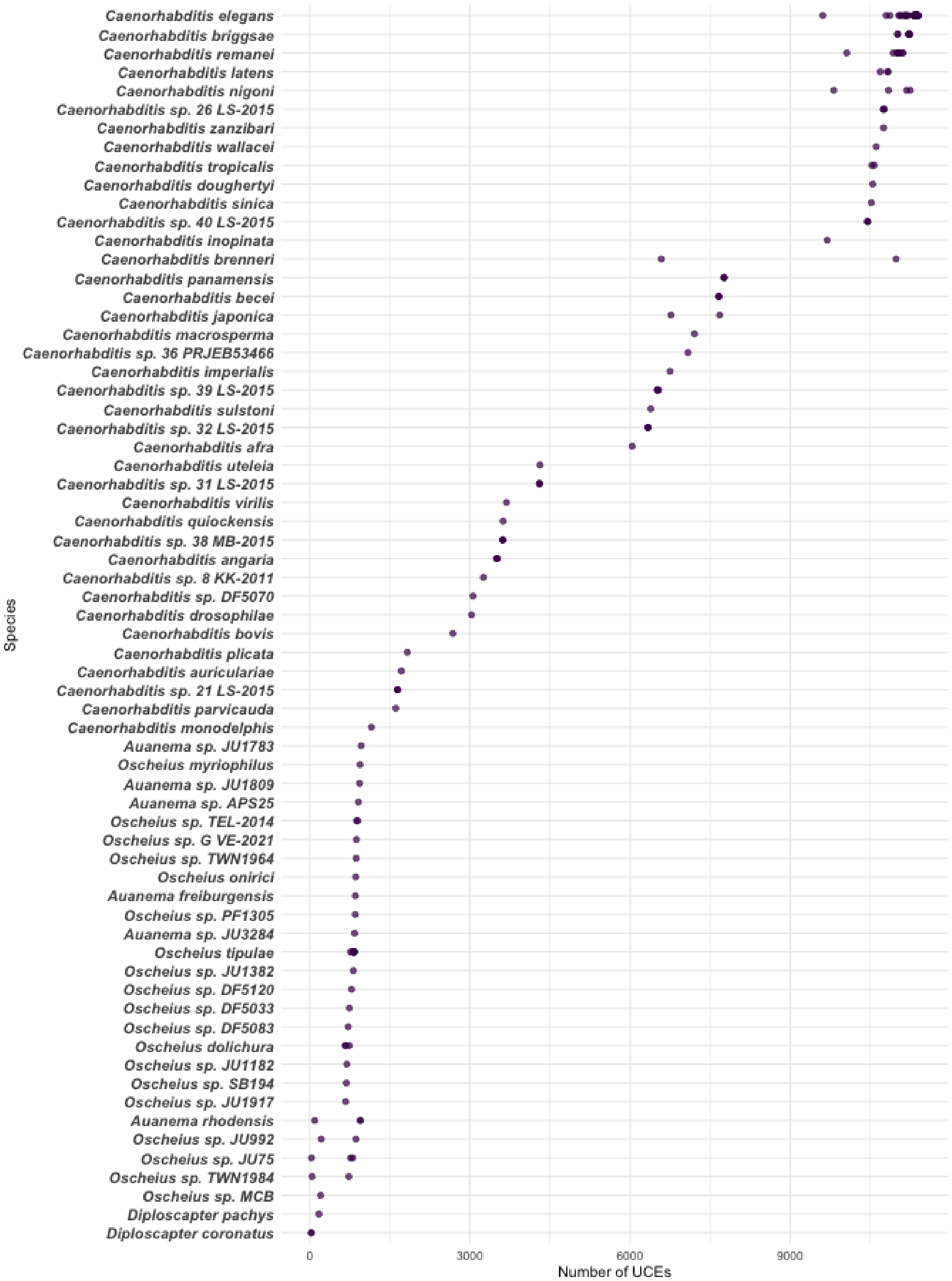

Figure 13: Number of UCEs per species in Rhabditidae. Species are sorted from lowest to highest UCE count.

A.3.1 UCEs shared across all the genera in the Rhabditidae and Panagrolaimidae family

Family	Total number	UCE name
Rhabditidae	50	uce.1269, uce.16032, uce.1524, uce.1260, uce.1673, uce.1892, uce.14291, uce.15873, uce.512, uce.16435, uce.19283, uce.7014, uce.15882, uce.262, uce.1190, uce.1271, uce.13410, uce.655, uce.1272, uce.14817, uce.424, uce.5519, uce.2159, uce.2212, uce.2407, uce.583, uce.1309, uce.4011, uce.16149, uce.6068, uce.2630, uce.731, uce.16271, uce.17033, uce.1326, uce.16433, uce.2401, uce.3013, uce.7820, uce.739, uce.1319, uce.15494, uce.18743, uce.2484, uce.15826, uce.737, uce.1739, uce.1905, uce.1984, uce.18176
Panagrolaimidae	84	uce.2078, uce.1795, uce.5994, uce.5306, uce.8657, uce.8674, uce.8695, uce.8786, uce.9007, uce.10095, uce.10105, uce.10196, uce.8702, uce.10397, uce.281, uce.284, uce.431, uce.459, uce.604, uce.618, uce.663, uce.9794, uce.7942, uce.4462, uce.6371, uce.6493, uce.7157, uce.5355, uce.5498, uce.5515, uce.4246, uce.9888, uce.9910, uce.4359, uce.4261, uce.10506, uce.10578, uce.7322, uce.7567, uce.7646, uce.7753, uce.7765, uce.6749, uce.10627, uce.6806, uce.6606, uce.7323, uce.8091, uce.8113, uce.8114, uce.8234, uce.4024, uce.4162, uce.5069, uce.8318, uce.8373, uce.8398, uce.8429, uce.9348, uce.9422, uce.9427, uce.4491, uce.4493, uce.4559, uce.4655, uce.5655, uce.5769, uce.5836, uce.5845, uce.9316, uce.4329, uce.6370, uce.7084, uce.323, uce.5837, uce.7309, uce.7996, uce.3, uce.194, uce.1316, uce.10496, uce.227, uce.6826, uce.5043

Table 13: Shared UCEs across all genera for each family.

A.4 Benchmark of Machine Learning Models on Rhabditidae Data

A comprehensive comparison of the models is provided through an AUC comparison plot, illustrating the relative performance of each approach, see Figure 14.

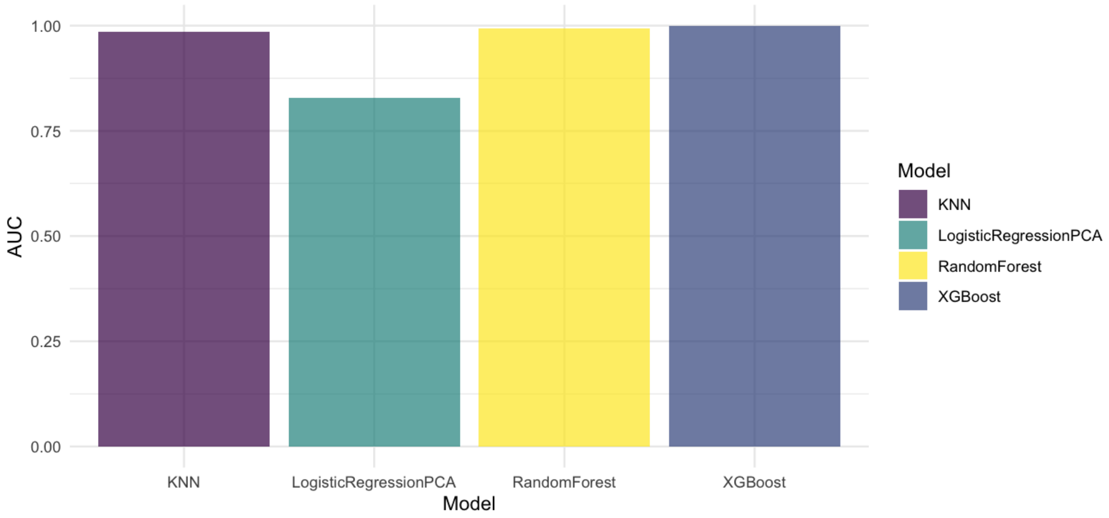

Figure 14: Comparison of AUC scores across ML models on Rhabditidae data.

Complete class-specific performance metrics are included, detailing sensitivity, specificity, precision, recall, and balanced accuracy. Additionally, confusion matrix heatmaps for each model offer visual insights into classification accuracy across all genera, see Figure 15.

Random Forest

```
Overall Statistics
               Accuracy : 0.8507
                 95% CI : (0.7426, 0.926)
    No Information Rate : 0.7164
    P-Value [Acc > NIR] : 0.007787
                  Kappa : 0.6819
 Mcnemar's Test P-Value : NA


Statistics by Class:
                      Sensitivity Specificity Pos Pred Value Neg Pred Value  Precision    Recall
Class: Auanema          1.0000000   1.0000000     1.00000000           1.00 1.00000000 1.0000000
Class: Caenorhabditis   0.9791667   1.0000000     1.00000000           0.95 1.00000000 0.9791667
Class: Diploscapter     1.0000000   0.8484848     0.09090909           1.00 0.09090909 1.0000000
Class: Oscheius         0.4375000   1.0000000     1.00000000           0.85 1.00000000 0.4375000

                             F1 Prevalence Detection Rate Detection Prevalence Balanced Accuracy
Class: Auanema        1.0000000 0.02985075     0.02985075           0.02985075         1.0000000
Class: Caenorhabditis 0.9894737 0.71641791     0.70149254           0.70149254         0.9895833
Class: Diploscapter   0.1666667 0.01492537     0.01492537           0.16417910         0.9242424
Class: Oscheius       0.6086957 0.23880597     0.10447761           0.10447761         0.7187500
```

Logistic Regression with PCA

```
Overall Statistics
               Accuracy : 0.9403
                 95% CI : (0.8541, 0.9835)
    No Information Rate : 0.7164
    P-Value [Acc > NIR] : 4.383e-06
                  Kappa : 0.8599
 Mcnemar's Test P-Value : NA


Statistics by Class:
                      Sensitivity Specificity Pos Pred Value Neg Pred Value Precision    Recall
Class: Auanema          0.0000000   1.0000000            NaN      0.9701493        NA 0.0000000
Class: Caenorhabditis   0.9791667   1.0000000            1.0      0.9500000       1.0 0.9791667
Class: Diploscapter     0.0000000   1.0000000            NaN      0.9850746        NA 0.0000000
Class: Oscheius         1.0000000   0.9215686            0.8      1.0000000       0.8 1.0000000

                             F1 Prevalence Detection Rate Detection Prevalence Balanced Accuracy
Class: Auanema               NA 0.02985075      0.0000000            0.0000000         0.5000000
Class: Caenorhabditis 0.9894737 0.71641791      0.7014925            0.7014925         0.9895833
Class: Diploscapter          NA 0.01492537      0.0000000            0.0000000         0.5000000
Class: Oscheius       0.8888889 0.23880597      0.2388060            0.2985075         0.9607843
```

k-Nearest Neighbors

```
Overall Statistics
               Accuracy : 0.9403
                 95% CI : (0.8541, 0.9835)
```

```
    No Information Rate : 0.7164
    P-Value [Acc > NIR] : 4.383e-06
                  Kappa : 0.8652
 Mcnemar's Test P-Value : NA


Statistics by Class:
                      Sensitivity Specificity Pos Pred Value Neg Pred Value Precision    Recall
Class: Auanema          1.0000000   1.0000000      1.0000000      1.0000000 1.0000000 1.0000000
Class: Caenorhabditis   0.9583333   1.0000000      1.0000000      0.9047619 1.0000000 0.9583333
Class: Diploscapter     0.0000000   0.9848485      0.0000000      0.9848485 0.0000000 0.0000000
Class: Oscheius         0.9375000   0.9411765      0.8333333      0.9795918 0.8333333 0.9375000

                             F1 Prevalence Detection Rate Detection Prevalence Balanced Accuracy
Class: Auanema        1.0000000 0.02985075     0.02985075           0.02985075         1.0000000
Class: Caenorhabditis 0.9787234 0.71641791     0.68656716           0.68656716         0.9791667
Class: Diploscapter         NaN 0.01492537     0.00000000           0.01492537         0.4924242
Class: Oscheius       0.8823529 0.23880597     0.22388060           0.26865672         0.9393382
```

XGBoost

```
Overall Statistics
               Accuracy : 0.9851
                 95% CI : (0.9196, 0.9996)
    No Information Rate : 0.7164
    P-Value [Acc > NIR] : 5.443e-09
                  Kappa : 0.9657
 Mcnemar's Test P-Value : NA


Statistics by Class:
                      Sensitivity Specificity Pos Pred Value Neg Pred Value Precision    Recall
Class: Auanema          1.0000000   1.0000000      1.0000000           1.00 1.0000000 1.0000000
Class: Caenorhabditis   0.9791667   1.0000000      1.0000000           0.95 1.0000000 0.9791667
Class: Diploscapter     1.0000000   1.0000000      1.0000000           1.00 1.0000000 1.0000000
Class: Oscheius         1.0000000   0.9803922      0.9411765           1.00 0.9411765 1.0000000

                             F1 Prevalence Detection Rate Detection Prevalence Balanced Accuracy
Class: Auanema        1.0000000 0.02985075     0.02985075           0.02985075         1.0000000
Class: Caenorhabditis 0.9894737 0.71641791     0.70149254           0.70149254         0.9895833
Class: Diploscapter   1.0000000 0.01492537     0.01492537           0.01492537         1.0000000
Class: Oscheius       0.9696970 0.23880597     0.23880597           0.25373134         0.9901961
```

A.4.1 Identification of important features using non-zero importance scores in XGBoost for Rhabditidae family

A cross-validation procedure was conducted to assess the impact of feature count on model performance. The process involved iteratively training the XGBoost model with increasing numbers of top-ranked features, as determined by their importance scores. Specifically, the model was trained and evaluated using feature subsets ranging from the single most important feature up to the top 20 features. For each iteration, the Area Under the Curve (AUC) was calculated using the 'multiclass.roc' function from the 'pROC' package, providing a measure of the model's predictive accuracy. The resulting AUC values were then plotted against the number of features used, generating a visual representation of the relationship between feature count and model performance.

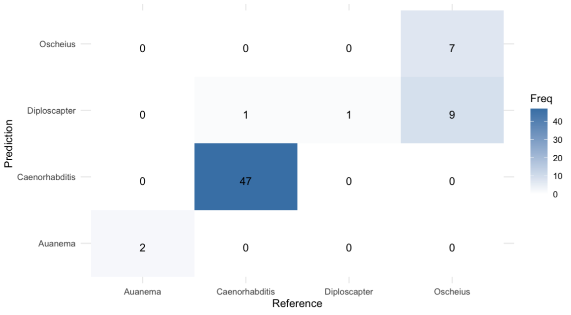

(a) Random Forest

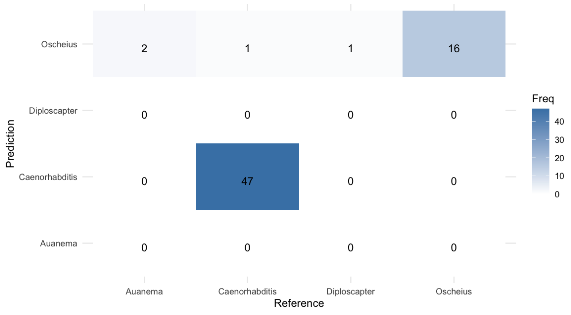

(b) Logistic Regression with PCA

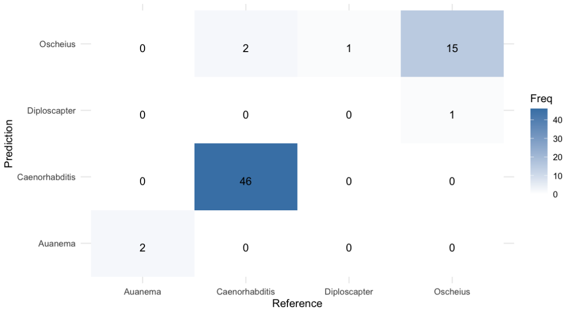

(c) k-Nearest Neighbors

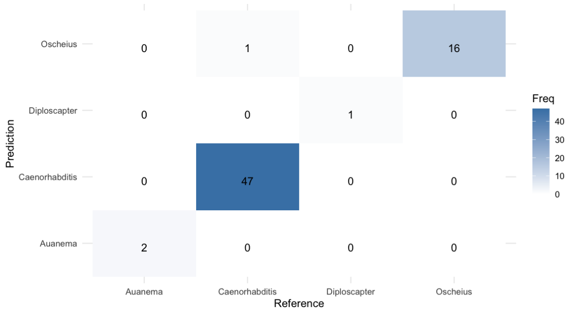

(d) XGBoost

Figure 15: Confusion matrix heatmaps for different ML models on Rhabditidae Data.

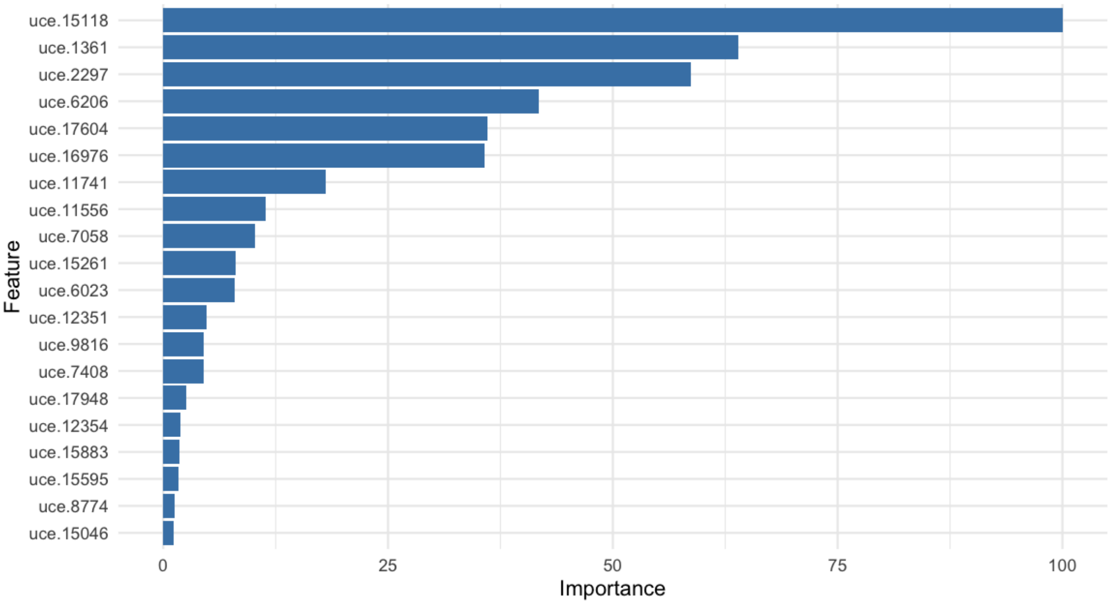

Figure 16: Top 20 features ranked by importance scores (XGBoost) for Rhabditidae family.

Table 14: Feature importance scores (XGBoost) on the Rhabditidae family, sorted in descending order.

#	Feature	Overall	#	Feature	Overall
1	uce.15118	100.00000000	24	uce.3117	0.57467362
2	uce.1361	63.98891640	25	uce.1508	0.55899331
3	uce.2297	58.70899293	26	uce.377	0.54091615
4	uce.6206	41.76429892	27	uce.6314	0.40157400
5	uce.17604	36.08823354	28	uce.9054	0.29854266
6	uce.16976	35.71085919	29	uce.7504	0.29501397
7	uce.11741	18.01152015	30	uce.15693	0.28641136
8	uce.11556	11.37746178	31	uce.1739	0.26911590
9	uce.7058	10.21175252	32	uce.4435	0.26010081
10	uce.15261	8.06170688	33	uce.14951	0.20215311
11	uce.6023	7.97789374	34	uce.739	0.19555710
12	uce.12351	4.82092405	35	uce.5314	0.17701833
13	uce.9816	4.52307700	36	uce.16080	0.17486536
14	uce.7408	4.45983387	37	uce.9712	0.16037217
15	uce.17948	2.56738332	38	uce.16977	0.14765256
16	uce.12354	1.90536817	39	uce.12716	0.13513669
17	uce.15883	1.84721198	40	uce.15269	0.12088749
18	uce.15595	1.69534444	41	uce.10818	0.11498949
19	uce.8774	1.26314020	42	uce.13654	0.07959777
20	uce.15046	1.13152980	43	uce.8269	0.06693109
21	uce.16507	1.08547413	44	uce.9042	0.04954661
22	uce.12197	0.96881894	45	uce.16032	0.03010273
23	uce.14879	0.88733092	46	uce.12732	0.02833252

A.5 Panagrolaimidae classification model performance

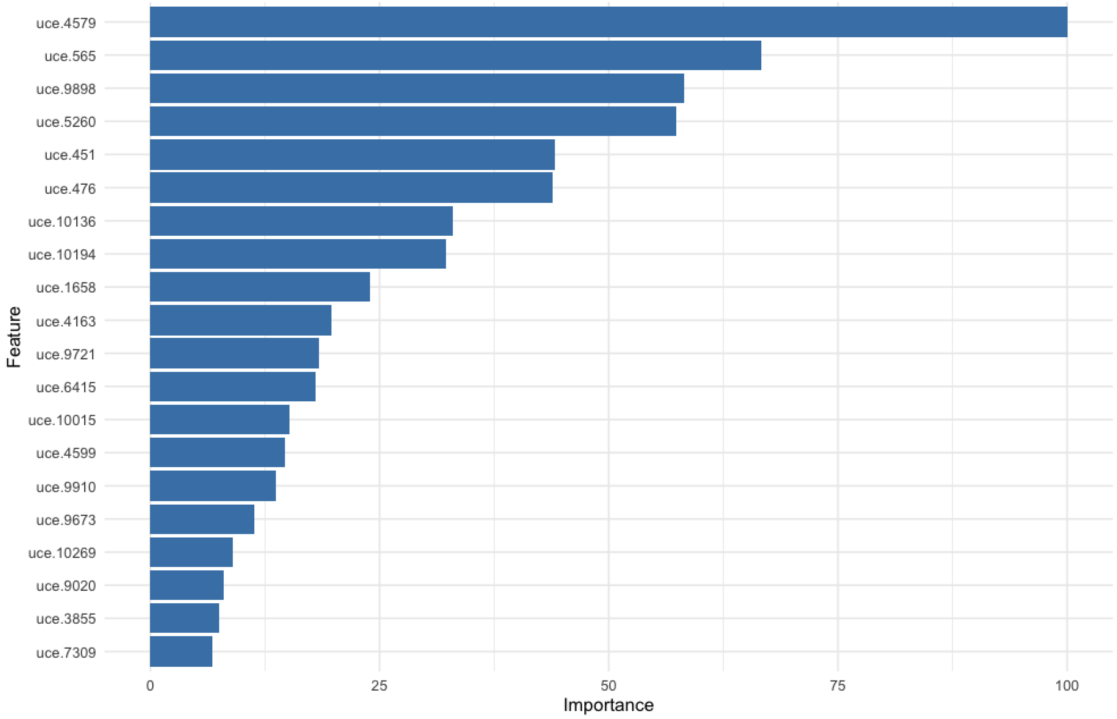

Figure 17: Top 20 features ranked by importance scores (XGBoost) for Panagrolaimidae family.

Table 15: Top UCE feature importance and their overall scores for the Panagrolaimidae family

#	Feature	Overall	#	Feature	Overall	#	Feature	Overall
1	uce.4579	100.000000	14	uce.4599	14.631350	27	uce.459	5.041798
2	uce.565	66.623360	15	uce.9910	13.704530	28	uce.215	3.982949
3	uce.9898	58.208410	16	uce.9673	11.354810	29	uce.7806	3.966508
4	uce.5260	57.338690	17	uce.10269	9.037717	30	uce.9218	3.224067
5	uce.451	44.179020	18	uce.9020	7.986509	31	uce.4381	3.149135
6	uce.476	43.890580	19	uce.3855	7.445594	32	uce.6949	2.629612
7	uce.10136	32.933190	20	uce.7309	6.782274	33	uce.8786	2.131640
8	uce.10194	32.206180	21	uce.7825	6.602921	34	uce.6827	2.059504
9	uce.1658	23.978340	22	uce.10532	6.213068	35	uce.7163	2.057336
10	uce.4163	19.712390	23	uce.194	6.027681	36	uce.6376	1.980775
11	uce.9721	18.340240	24	uce.8785	5.904270	37	uce.9291	1.826193
12	uce.6415	18.004650	25	uce.5243	5.803961	38	uce.4230	1.572708
13	uce.10015	15.175680	26	uce.8674	5.418900	39	uce.323	1.553780

XGBoost - full features Panagrolaimidae

```
Overall Statistics
               Accuracy : 0.9412
                 95% CI : (0.7131, 0.9985)
    No Information Rate : 0.7059
    P-Value [Acc > NIR] : 0.02168
                  Kappa : 0.8759
```

```
 Mcnemar's Test P-Value : NA


Statistics by Class:
                        Sensitivity Specificity Pos Pred Value Neg Pred Value Precision Recall  F1
Class: Acrobeloides               1   0.9333333      0.6666667      1.0000000 0.6666667      1 0.8
Class: Halicephalobus             1   1.0000000      1.0000000      1.0000000 1.0000000      1 1.0
Class: Panagrellus                1   1.0000000      1.0000000      1.0000000 1.0000000      1 1.0
Class: Panagrolaimus              1   1.0000000      1.0000000      1.0000000 1.0000000      1 1.0
Class: Propanagrolaimus           0   1.0000000            NaN      0.9411765        NA      0  NA

                        Prevalence Detection Rate Detection Prevalence Balanced Accuracy
Class: Acrobeloides     0.11764706     0.11764706           0.17647059         0.9666667
Class: Halicephalobus   0.05882353     0.05882353           0.05882353         1.0000000
Class: Panagrellus      0.05882353     0.05882353           0.05882353         1.0000000
Class: Panagrolaimus    0.70588235     0.70588235           0.70588235         1.0000000
Class: Propanagrolaimus 0.05882353     0.00000000           0.00000000         0.5000000
```

XGBoost - feature importance Panagrolaimidae

```
Overall Statistics
               Accuracy : 0.9412
                 95% CI : (0.7131, 0.9985)
    No Information Rate : 0.7059
    P-Value [Acc > NIR] : 0.02168
                  Kappa : 0.8859
 Mcnemar's Test P-Value : NA


Statistics by Class:
                        Sensitivity Specificity Pos Pred Value Neg Pred Value Precision    Recall
Class: Acrobeloides       1.0000000      1.0000            1.0      1.0000000       1.0 1.0000000
Class: Halicephalobus     1.0000000      0.9375            0.5      1.0000000       0.5 1.0000000
Class: Panagrellus        1.0000000      1.0000            1.0      1.0000000       1.0 1.0000000
Class: Panagrolaimus      0.9166667      1.0000            1.0      0.8333333       1.0 0.9166667
Class: Propanagrolaimus   1.0000000      1.0000            1.0      1.0000000       1.0 1.0000000

                               F1 Prevalence Detection Rate Detection Prevalence Balanced Accuracy
Class: Acrobeloides     1.0000000 0.11764706     0.11764706           0.11764706         1.0000000
Class: Halicephalobus   0.6666667 0.05882353     0.05882353           0.11764706         0.9687500
Class: Panagrellus      1.0000000 0.05882353     0.05882353           0.05882353         1.0000000
Class: Panagrolaimus    0.9565217 0.70588235     0.64705882           0.64705882         0.9583333
Class: Propanagrolaimus 1.0000000 0.05882353     0.05882353           0.05882353         1.0000000
```

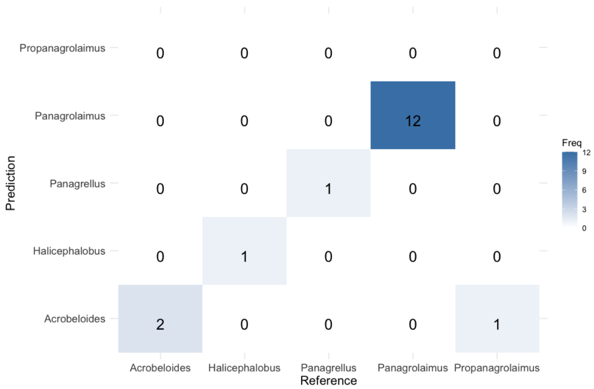

(a) Panagrolaimidae ML model created with with full UCE features

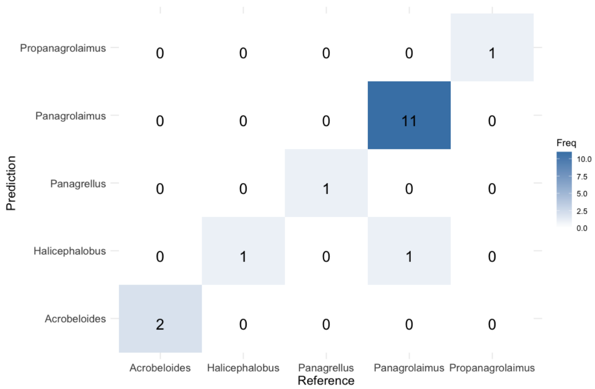

(b) Panagrolaimidae ML model created with Important features selected

Figure 18: Confusion matrices in Panagrolaimidae

A.6 Analysis of Common UCEs

Rhabditidae UCE Analysis Analysis of Ultra-Conserved Elements (UCEs) in Rhabditidae was performed to understand the genetic conservation and predictive features within this nematode family. UCEs were categorized into three groups: (1) UCEs shared across all analyzed species, (2) the most frequently observed UCEs, and (3) UCEs identified as important features by the XGBoost machine learning model. This analysis aims to highlight UCEs with potential biological significance by identifying overlaps between these groups.

UCE	Groups where it appears
uce.512	Shared, Frequent
uce.655	Shared, Frequent
uce.4011	Shared, Frequent
uce.1892	Shared, Frequent
uce.739	Shared, XGBoost
uce.1739	Shared, XGBoost
uce.16032	Shared, XGBoost
uce.13654	Frequent, XGBoost
uce.16977	Frequent, XGBoost
uce.7408	Frequent, XGBoost

Table 16: Summary of UCEs shared across Rhabditidae, grouped by their presence in Shared in all species (Shared), the most (Frequent), and the Feature selection in the XGBoost model (XGBoost) categories.

The UCEs shared between two groups offer valuable insights. For example, UCEs shared between "Shared by all species" and "Most Frequent" (uce.512, uce.655, uce.4011, uce.1892) indicate highly conserved and recurrent regions within Rhabditidae. UCEs shared between "Shared by all species" and XGBoost (uce.739, uce.1739, uce.16032) or "Most Frequent" and XGBoost (uce.13654, uce.16977, uce.7408) highlight regions important for species classification and differentiation. No UCEs are present in all three groups.

Panagrolaimidae UCE Analysis Analysis of Ultra-Conserved Elements (UCEs) in Panagrolaimidae was performed to understand the genetic conservation and predictive features within this nematode family. The UCEs were categorized into three groups: (1) UCEs shared across all analyzed species, (2) the 100 most frequently observed UCEs, and (3) UCEs identified as important features by the XGBoost machine learning model. This analysis aims to highlight UCEs with potential biological significance by identifying overlaps between these groups.

The UCEs found in all three groups (uce.9910, uce.194, uce.8674, uce.459, uce.8786, uce.323) are particularly significant as they represent regions that are not only conserved across all species but also frequently observed and identified as important predictors by the XGBoost model. This suggests these UCEs could play crucial roles in the fundamental biology and evolution of Panagrolaimidae. The UCEs shared between two groups also offer valuable insights. For example, UCEs shared between XGBoost and Most Frequent may indicate regions that are highly informative for species classification, while those shared between XGBoost and Shared by all species might highlight regions under strong selective pressure.

UCE	Groups where it appears
uce.9910	Shared, Frequent, XGBoost
uce.194	Shared, Frequent, XGBoost
uce.8674	Shared, Frequent, XGBoost
uce.459	Shared, Frequent, XGBoost
uce.8786	Shared, Frequent, XGBoost
uce.323	Shared, Frequent, XGBoost
uce.10196	Shared, XGBoost
uce.5837	Shared, XGBoost
uce.6806	Shared, XGBoost
uce.7309	Shared, XGBoost
uce.9348	Shared, XGBoost
uce.10095	Shared, Frequent
uce.10105	Shared, Frequent
uce.10397	Shared, Frequent
uce.1316	Shared, Frequent
uce.1795	Shared, Frequent
uce.281	Shared, Frequent
uce.4162	Shared, Frequent
uce.431	Shared, Frequent
uce.4329	Shared, Frequent
uce.4462	Shared, Frequent
uce.4491	Shared, Frequent
uce.5306	Shared, Frequent
uce.5355	Shared, Frequent
uce.5498	Shared, Frequent
uce.5515	Shared, Frequent
uce.604	Shared, Frequent
uce.618	Shared, Frequent
uce.7157	Shared, Frequent
uce.7646	Shared, Frequent
uce.7753	Shared, Frequent
uce.7765	Shared, Frequent
uce.8373	Shared, Frequent
uce.8702	Shared, Frequent
uce.9422	Shared, Frequent
uce.10015	Frequent, XGBoost
uce.10136	Frequent, XGBoost
uce.215	Frequent, XGBoost
uce.4163	Frequent, XGBoost
uce.4381	Frequent, XGBoost
uce.451	Frequent, XGBoost
uce.5260	Frequent, XGBoost
uce.574	Frequent, XGBoost
uce.9218	Frequent, XGBoost
uce.9721	Frequent, XGBoost

Table 17: Summary of UCEs shared across Panagrolaimidae, grouped by their presence in Shared in all species (Shared), the most (Frequent), and the Feature selection in the XGBoost model (XGBoost) categories.
